# One-dimensional hydrodynamic simulations of low convergence ratio direct-drive inertial confinement fusion implosions

**DOI:** 10.1098/rsta.2020.0224

**Published:** 2020-12-07

**Authors:** R. W. Paddock, H. Martin, R. T. Ruskov, R. H. H. Scott, W. Garbett, B. M. Haines, A. B. Zylstra, R. Aboushelbaya, M. W. Mayr, B. T. Spiers, R. H. W. Wang, P. A. Norreys

**Affiliations:** 1 Clarendon Laboratory, University of Oxford, Oxford, UK; 2 University College, University of Oxford, Oxford, UK; 3 Central Laser Facility, STFC, Rutherford Appleton Laboratory, Didcot, UK; 4 AWE plc, Aldermaston, Reading, Berkshire RG7 4PR, UK; 5 Los Alamos National Laboratory, MS T087, Los Alamos, NM 87545, USA; 6 Lawrence Livermore National Laboratory, Livermore, CA 94550, USA

**Keywords:** inertial confinement fusion, direct drive, convergence ratio, hydrodynamic instabilities, high gain

## Abstract

Indirect drive inertial confinement fusion experiments with convergence ratios below 17 have been previously shown to be less susceptible to Rayleigh–Taylor hydrodynamic instabilities, making this regime highly interesting for fusion science. Additional limitations imposed on the implosion velocity, in-flight aspect ratio and applied laser power aim to further reduce instability growth, resulting in a new regime where performance can be well represented by one-dimensional (1D) hydrodynamic simulations. A simulation campaign was performed using the 1D radiation-hydrodynamics code HYADES to investigate the performance that could be achieved using direct-drive implosions of liquid layer capsules, over a range of relevant energies. Results include potential gains of 0.19 on LMJ-scale systems and 0.75 on NIF-scale systems, and a reactor-level gain of 54 for an 8.5 MJ implosion. While the use of 1D simulations limits the accuracy of these results, they indicate a sufficiently high level of performance to warrant further investigations and verification of this new low-instability regime. This potentially suggests an attractive new approach to fusion energy.

This article is part of a discussion meeting issue ‘Prospects for high gain inertial fusion energy (part 2)’.

## 1. Introduction

Inertial confinement fusion (ICF) experiments, through extreme compression of deuterium–tritium (DT) fuel capsules, produce some of the most extreme conditions ever seen on Earth. By recreating the conditions present in stellar interiors the fusion of deuterium and tritium ions becomes possible, resulting in the release of energy. The ultimate goal of ICF research is to achieve net energy output from these experiments, enabling the generation of clean and sustainable electricity in ICF power stations. Yet despite substantial progress, attempts to achieve ignition (the point at which fusion self-heating balances losses and becomes self-sustaining) have so far been unsuccessful [1], and research efforts to achieve break-even remain ongoing [2].

The compression of the fuel capsule in an ICF experiment can be quantified using the convergence ratio, which is defined in this paper by

1.1 $$\text{CR}=R_{\text{I}}/R_{\text{HS}},$$

where *R*_I_ is the initial radius of the interior edge of the plastic capsule shell, and *R*_HS_ is the minimum radius of the hotspot (i.e. the hotspot radius at the time of maximum compression). In order to achieve fusion conditions, typical ICF experiments aim to compress the fuel as much as possible; in the National Ignition Campaign (NIC) at the National Ignition Facility (NIF), convergence ratios in the range of 30 < CR < 40 were targeted [3]. However, this resulted in the onset of significant hydrodynamic instabilities, which meant that fusion performance was significantly worse than expected from the campaign based on simulations. These instabilities were a key reason the NIC was unsuccessful in achieving ignition (along with fill tube, tent and capsule imperfections, all of which are also exacerbated by high convergence ratio), and continue to be a major challenge in ICF experiments [4]. Further discussion of the significance of convergence ratio will follow in §2a.

However, experiments operating at lower convergence ratios have observed significant reduction in hydrodynamic instabilities, and performance that is much closer to one-dimensional (this is discussed in more detail in §2b). This paper follows work by Olson *et al.* [5] and Zylstra *et al.* [6], who observed this on three NIF shots in the 12 < CR < 19 range. It was found that experiments operating at lower CR values in this range show good agreement with simulations, suggesting a region of phase space which is well described by current codes and where instabilities are of low significance. This work therefore aims to investigate this regime, and to demonstrate through a 1D simulation campaign that high gain and good performance can be achieved at low convergence ratio using the direct-drive approach.

It is important to restrict the focus of this campaign to a region of phase space where 1D simulations are physically meaningful, which means working in a regime where both hydrodynamic and parametric instabilities are minimized. Along with operating at low convergence ratio, this is achieved by selecting upper permissible limits on implosion velocity, in-flight aspect ratio and on-target intensity. By considering only the results of simulations which satisfy all four of these criteria, the campaign is limited to a regime where the effect of instabilities should be small and where the 1D simulation code should provide reasonable estimates of the experimental performance. The purpose of this paper is not to suggest that the specific gains that these simulations predict are actually obtainable experimentally. For this to be the case, more detailed simulations in higher dimensions would be required. The gains presented are also not in themselves particularly remarkable, with much higher gains being regularly reported for simulation work. Rather, the notable and novel aspect of this work is in its identification of a regime where instability growth is expected to be low, and the fact that 1D simulations suggest that reasonable gain is possible in such a regime. The 1D simulations are intended only to provide an indication of performance, but it is believed that this initial estimate is sufficiently high to warrant further investigation of this regime and approach.

The inability of 1D simulation codes to provide an accurate predictive capability is a significant obstacle to achieving ignition, and attempts to mitigate this issue (aside from this paper’s approach of operating in a regime where these capabilities are better) are under development. A notable example of research efforts to address this problem was presented by Gopalaswamy *et al*. [7], who used a statistical approach to find relationships between simulated code outputs and existing experimental data in order to better predict future performance. Experiments at the OMEGA facility on the basis of their predictions allowed them to generate an impressive neutron yield of 1.6E14, which would correspond to 500 kJ of fusion energy on the NIF when hydrodynamic scaling is applied.

As additional motivation for studying this topic, low convergence ratio implosions are ideal for use with other new developments in ICF experiments. For example, Ratan *et al.* [8] have recently proposed a novel auxiliary heating scheme, based on crossing two relativistic electron beams in the hotspot of an ICF implosion [8]. This would lead to an increase in electron temperature in the hotspot, which would rapidly equilibrate and raise the ion temperature, thus increasing the neutron yield. Such a scheme would require a large central hotspot, such as that produced in a low convergence ratio implosion [9]. The possibility of augmenting low CR implosions with such schemes (and the use of low CR implosions in assisting the development of such schemes) provides another compelling reason to investigate low convergence ratio implosions.

Convergence ratios of ICF capsules are typically controlled by varying the DT vapour pressure within the capsule, which is in turn controlled through varying the capsule temperature [10]. The vapour pressures necessary to facilitate the convergence ratios discussed in this work require temperatures greater than the melting point of DT ice, which makes the use of conventional DT ice layers impossible. Instead, this work describes the use of liquid DT layers. However, liquid layers have been observed to sag due to the effects of gravity, resulting in increased thickness at the bottom of the capsule [11]. There are a few ways to prevent this effect, and to produce uniform liquid layers. Firstly, a small thermal gradient can be established across the capsule to compensate for the effect of gravity and result in a uniform layer [12]. This has been successfully implemented experimentally, using low power Ar lasers to provide the heating [13,14]. Alternatively, low-density CH foam can be wetted with liquid DT to produce a uniform liquid layer over the foam structure. This technique (which was used in the work of Olson and Zylstra) is simpler to implement, but the presence of the foam must then be accounted for. In this case, the liquid layer can only be described using a pure DT equation of state (EOS) if the unwetted foam density is below approximately 10 mg cm^−3^ (Haines BM, Zylstra A 2020 Personal Communication); this is significantly lower than the 25 mg cm^−3^ foam density that can currently survive the wetting process [15]. For this reason, further study of foams and development of the manufacturing techniques are encouraged (along with the collection of EOS data for wetted foams at currently obtainable densities). The simulations presented in this work (which describe the liquid layer using the pure DT EOS) can therefore be viewed from a number of perspectives: as a simulation of a pure liquid layer capsule (which could be achieved using the thermal gradient technique), as an optimistic first approximation of what could be achieved using current wetted foam layers, or as a simulation of performance that could potentially be achieved in the future using hypothetical wetted foam capsules where the foam density is below the aforementioned threshold value.

## 2. Background and theory

### (a) Ignition, performance and convergence ratio

The ignition threshold factor (ITF) is a key equation for capsule designers, providing a measure of proximity to ignition for a given implosion based on a number of variables essential to hotspot formation [16]. Margin (or proximity to ignition) is useful because measured quantities such as yield can vary rapidly around ignition; a barely igniting capsule may nonetheless have a high yield, but a minor change may result in it failing to ignite and thus the yield dropping suddenly and substantially [17]. The ITF is useful for this paper to highlight the key variables for achieving ignition; further discussion of the use of ITF and other performance metrics for assessing implosion performance and robustness can be found in [17]. The ITF is given by

2.1 $$\text{ITF}=I_{0}\left(\frac{M_{\text{DT}}}{M_{0}}\right){\left(\frac{v}{v_{0}}\right)}^{8}{\left(\frac{\alpha }{\alpha_{0}}\right)}^{-4}{\left(1-1.2\frac{\Delta R_{\text{HS}}^{\text{K-wtd}}}{R_{\text{HS}}}\right)}^{4}{\left(\frac{M_{\text{clean}}}{M_{\text{DT}}}\right)}^{0.5}(1-P_{\text{HS}}),$$

where *I*_0_ is the ITF of a baseline optimized 1D implosion, *M*_DT_ is the fuel mass of the capsule, *v* is the implosion velocity, *α* is the adiabat of the main fuel, $\Delta R_{\text{HS}}^{\text{K-wtd}}$ is the Kishony-weighted RMS deviation from the the average hotspot radius *R*_HS_, *M*_clean_ is the DT mass not contaminated by mixing, and *P*_HS_ is a measure of the purity of the hotspot. The terms *M*_0_, *v*_0_ and *α*_0_ are nominal values for the target under consideration.

The implosion velocity appears in the ITF equation to the eighth power, clearly demonstrating the significance of this parameter. The implosion velocity is also linked to the convergence ratio, by the equation

2.2 $$\mathrm{CR}∼{\left(\frac{v^{2}\cdot \rho_{{\;}_{d}}}{p_{{\;}_{d}}}\right)}^{1/3},$$

where *ρ*_d_ and *p*_d_ are the in-flight density and in-flight pressure of the shell recorded just before it begins to decelerate [18]. From this, it can be easily seen that ITF is proportional to the twelfth power of convergence ratio, demonstrating clearly how intrinsic the convergence ratio is to achieving ignition and why previous work has aimed to maximize this value. CR also influences ITF through the hotspot perturbation term,

2.3 $$\frac{\Delta R_{\text{HS}}}{R_{\text{HS}}}=\frac{\Delta a}{a}(\text{CR}-1),$$

where *a* is the acceleration and Δ*a* is the acceleration perturbation [19]. This again demonstrates the link between convergence and ITF, and also demonstrates how the convergence ratio is tied to hotspot asymmetry. Other expressions linking implosion velocity to hotspot temperature, *T*_HS_ ∝ *v*^2^, and neutron yield, *Y* ∝ *v*^10^, further demonstrate the significance of CR on ICF performance [20].

While the ITF is often used in capsule design, evaluating performance is more often achieved by measuring the areal density *ρR* and ion temperature *T* achieved in the hotspot and shell. This is common for two major reasons. Firstly, these parameters are more similar to those in the Lawson criterion [21], and appear directly in the generalized Lawson criterion used to compare ICF progress with magnetic confinement fusion [22]. Secondly, these quantities can be obtained far more easily from experimental data [23]. Various models exist to give an indication of the conditions required for ignition to occur, such as the one derived by Cheng *et al.*

2.4 $${\left(\rho R\right)}_{\text{HS}}\geq 4\frac{\kappa_{\text{C}}}{f_{\text{T}}}{\left(\frac{T}{\text{keV}}\right)}^{-2.5}\;{\text{g cm}}^{-2},$$

where *κ*_C_ ≃ 5.514 is a constant depending on the power law approximation for DT reactivity, and $f_{\text{T}}\propto \sqrt{\rho_{\text{p}}/\rho_{\text{HS}}}$ is a tamping factor (typically assumed to be 1 for basic estimates of ignition threshold) [24]. In this case, both the temperature and areal density refer to those of the hotspot. This describes an ignition threshold curve, with both values depending significantly on the degree of convergence.

The ideal ignition temperature (or temperature at which the fusion energy produced is greater than the radiation losses) is further increased when different ions are present in the plasma. Bremmstrahlung emission scales with atomic number as *Z*^2^, and so the presence of heavier ions contributes to increased radiation losses and higher ignition thresholds [25]. In wetted foam implosions, the presence of carbon ions in the central plasma (from the CH foam) can therefore result in higher ignition temperatures than would otherwise be expected. A worst-case estimate of this effect can be calculated by considering a plasma where the atomic composition equals that of the foam layer. With current manufacturing techniques, a 250 mg cm^−3^ wetted foam layer will contain 25 mg cm^−3^ of CH foam, giving an atomic fraction for carbon of approximately 2% [26]. A plasma with this atomic composition has an ideal ignition temperature of approximately 5.2 keV, compared to approximately 4.4 keV when no carbon is present. This demonstrates that the ignition temperature will be increased by the presence of carbon, and may be higher than predicted by equation (2.4). Despite the fact that this estimate does not account for the actual hotspot composition and likely overestimates the carbon content, it is likely that it still an underestimate of the temperature at which a capsule would actually achieve ignition. This is because it is an ideal estimate where only radiation losses due to Bremmstrahlung are considered, and in reality other processes such as conduction, hydrodynamic effects and alpha particles losses (i.e. accounting for the fact that not all alpha particles are absorbed, and so some of the generated energy is lost from the system) also lead to an increase in ignition temperature.

While the hotspot conditions are key for determining if the capsule ignites, the main fuel conditions are also required in order to determine the energy produced. The burn up fraction of a capsule describes how much of the DT fuel is actually burnt (i.e. undergoes fusion), and can be estimated by [27]

2.5 $$\Phi =\frac{\rho R}{6.3+\rho R}.$$

Using this equation, it is evident that the shell areal density must be around 3 g cm^−2^ in order to achieve a burn up factor of around 0.3. This expression gives the maximum possible burn up for this shell density, and is obviously dependent on the conditions in the hotspot.

### (b) Low convergence ratio

Numerous authors have identified that ICF experiments performed at lower convergence ratios can show improved agreement with simulations; a (by no means complete) selection can be found in references [28–35]. Such a trend was evident as far back as 1980 [36] when it was observed that lower initial aspect ratios (thicker shells, and as such lower implosion velocities and lower convergence ratios) resulted in more 1D-like performance; however, the laser energies available at the time made the pursuit of high gain at low convergence ratios unfeasible. This trend was again observed recently in the work performed by Olson *et al.*, looking at indirect drive shots on wetted foam capsules on the NIF [5]. The NIF shot N160421 (with CR of 12) was compared with both 2D and 1D simulations (with a package to account for mix included), and good agreement was observed in both cases. The ability to well describe this shot with a 1D code suggests performance that is close to one-dimensional.

Zylstra *et al.* later analysed three liquid layer shots (including N160421) [6]. The three shots, with CR of 12, 17 and 19 (calculated using the same definition given in equation (1.1)) were compared with 2D simulations performed using the codes HYDRA and XRAGE. They found that while the experimental neutron yield for the CR 12 capsule was in the range of 0.7–0.9 of the simulated result, and was around 0.6–0.7 for the CR 17 capsule, this dropped to around 0.3 for the CR 19 capsule. Considering these two works together, two clear conclusions can be drawn. Firstly, it is clear that the performance at the low end of this CR range is sufficiently one-dimensional that such implosions can be well described by 1D simulations. It is reasonable that this is the case, as reducing convergence ratio is commonly acknowledged as a way of reducing the growth of hydrodynamic instabilities [37]. Secondly, it is also clear that in the 12 < CR < 19 range identified by these papers, there is a gradual decrease in the level of agreement between simulations and experiments as convergence ratio increases, which becomes quite dramatic at the highest CR.

Similar behaviours have also been reported in simulation work, where performance has again been found to be increasingly one-dimensional at low convergence ratios. Two-dimensional simulations performed in preparation for a wetted foam experimental campaign at the NIF observed that the ratio of 2D to 1D yield was in the range of 95–100% for low convergence ratio (CR 15), but was significantly reduced for higher convergence ratios [38], which shows agreement with the experimental work previously discussed. This contributed to the hypothesis that ‘the predictive capability of hot spot formation is robust and 1D-like for a relatively low CR hot spot (CR 15), but will become less reliable as hot spot CR is increased to CR > 20’. This is further supported by simulations performed in [26], where 2D simulations were used to measure the fraction of 1D yield achieved as a function of the Legendre *P*_4_ flux asymmetry perturbation parameter, and it was again observed that the fraction is much higher at lower convergence ratios.

While these works have focused on indirect drive, direct-drive experiments have also observed behaviour that is more 1D-like at low CR. Direct-drive experiments on OMEGA looking at shots with adiabats greater than 3.5 observed that the hotspot pressure and compressed areal density (the focus of the study) measured experimentally showed much closer agreement with 1D simulations for convergence ratios below 17 [39]. This level of agreement was quite high; the hotspot pressure averaged over the measured neutron rate was found to be about 90% of that produced by the 1D simulations. A rapid decrease in agreement was observed above CR = 17, which agrees with the previously discussed findings presented by Zylstra *et al*. As well as using direct rather than indirect drive, the work at OMEGA also used conventional DT ice layers rather than wetted foams. The fact that a similar trend was observed in the OMEGA work suggests therefore that the observations made by Olson *et al.* and Zylstra *et al.* are applicable more broadly than to just indirectly driven wetted foam capsules, and also likely applies for direct-drive ICF.

However, it is also clear from §2a that 1D performance and yield increase with increasing CR. There is therefore a compromise to be made between improved idealized 1D performance, and increased agreement between simulation and experiment. Based on the results in [5,6], this work will use an upper limit of 16 for the convergence ratio (the first criterion which all simulations in the simulation campaign are tested against). This sits firmly in the region where the agreement is reasonably good, but enables a much greater level of 1D performance than would be possible using the lowest CR values in this range.

### (c) Instabilities

There are two key classes of instability that affect ICF experiments. Hydrodynamic instabilities are largely responsible for the mixing of cold fuel into the hotspot and the mixing of ablator material into the fuel. Both of these effects reduce performance in ICF experiments [40]. It is these instabilities that are reduced by operating at lower convergence ratios. One of the most significant hydrodynamic instabilities is the Rayleigh–Taylor (RT) instability. RT instabilities occur when a low-density fluid is accelerated into a higher density one, and result in the rapid growth of small perturbations at the boundary. This can ultimately lead to break up of the layers, and mixing of the two fluids. The (ablation stabilized) growth rate is given by

2.6 $$\gamma =\sqrt{\frac{ak}{1+kL}}-\beta ku_{\text{a}},$$

where *a* is acceleration, *k* is the wavenumber of the perturbation, *L* is the minimum density scale length at the ablation front, *β* is a numerically determined coefficient, and *u*_a_ is the ablation velocity [25]. Other hydrodynamic instabilities such as the Richtmyer–Meshkov and Kelvin–Helmholtz instabilities have different mechanisms, but all ultimately can result in the growth of perturbations at boundaries between different fluids.

The effect of convergence ratio on instability growth can be seen from this equation. For a given initial capsule size, increasing convergence means either the capsule is compressed faster, or the capsule is compressed over a longer period of time. In the first case, the acceleration must be increased, while in the second case, the growth rate is applied over a longer period; it is clear that either possibility leads to more instability growth. However, it is also evident from equation (2.6) that other factors can also influence instability growth, and should be controlled if instabilities are to be minimized.

This work imposes an upper limit of 400 km s^−1^ to implosion velocity, which is in part motivated by limiting instability growth. The implosion velocity is obviously linked to the acceleration, and so limiting the velocity indirectly limits the acceleration to some extent (although obviously, it does not account for the time dependence), and thus again helps to reduce instability growth. If the constraint were chosen solely for this reason, limiting the maximum acceleration would be a better choice. However, implosion velocity is a widely quoted and used quantity for ICF implosions (unlike acceleration, and as seen by its appearance in the ITF in §2a), making it more useful to measure and compare with other work. Setting a constraint on the implosion velocity therefore can also be used to ensure that the design is feasible. The value of 400 km s^−1^ is characteristic of the highest velocities commonly used in other ignition designs [41], and is chosen to ensure that the design is realistic (rather than corresponding to a particular cut-off for some behaviour).

A measure of the minimum density scale length can be obtained using the in-flight aspect ratio (IFAR), which measures the ratio of shell radius to shell thickness. IFAR is defined as

2.7 $$\text{IFAR}=\frac{R_{\text{shell}}}{t_{\text{shell}}},$$

where *R*_shell_ and *t*_shell_ are the shell radius and thickness respectively, as measured when the shell outer radius is at two-thirds of the initial radius [41]. The minimum density scale length is inversely proportional to the density gradient at the ablation front, and so a thin shell (with a high-density gradient) results in a low scale length. It is clear that IFAR scales inversely to scale length (since a thin shell would result in a high IFAR), and as such hydrodynamic instabilities increase with increasing IFAR, as expected from equation (2.6). Through the use of some simple approximations, equation (2.6) can be converted into a form which highlights very clearly the significance of IFAR. As explained by Atzeni and Meyer-Ter-Vehn [25], by making some simple approximations for the different variables (including assuming constant acceleration over some fraction of the capsule radius), the equation

2.8 $$G_{l}=exp\left[\alpha_{1}{\left(\frac{l}{l+\alpha_{2}\cdot l\cdot {\left(\text{IFAR}\right)}^{-1}}\right)}^{1/2}-\alpha_{3}\cdot l\cdot {\left(\text{IFAR}\right)}^{-1}\right],$$

can be derived. *l* is the spherical harmonic mode number, and the three *α* values are numerical constants. This expression describes RT instability growth at the capsule outer surface during the acceleration of the shell inwards, but equivalent expressions can be derived describing feed through of this growth to the inner surface of the shell, and to describe the growth at this inner surface during deceleration; both of these also depend on IFAR [25]. The fact that these assumptions lead to formulae for the instability growth that depend only on IFAR for a given mode number demonstrates the importance of this parameter for controlling instabilities. An upper limit of 30 has been selected for IFAR in this work; this value is typical of the acceptable IFAR when using this equation, as capsules with higher IFAR’s have been observed to break up over the course of the implosion [19]. However, recent research has found that reducing the IFAR further still may be necessary to prevent instability growth [42,43], suggesting that further investigation of this regime may find reducing this limit to be beneficial.

Adiabat is also known to have an effect on instability growth, as lower fuel adiabats lead to lower ablation velocities and lower density scale lengths, which results in larger instability growth [44]. This is again a compromise, as reducing fuel adiabat results in increased 1D gain. Varying the adiabat is less simple than varying the other parameters mentioned, as it is primarily achieved by changing the relative amplitudes of the laser pulse profile which in turn requires significant changes to the laser timings. This work does not intend to vary the laser profiles in this way, as doing so is time- and labour-intensive (requiring the laser timings to be reoptimized), and makes the optimization campaign less manageable by adding many more variables. With the other three criteria providing stringent restrictions on instability growth, it was therefore decided that an adiabat limit would not be implemented in this initial investigation. However, research such as that by Goncharov *et al.* highlights the importance of the adiabat for instability growth (showing how IFAR and adiabat together can be significant in achieving 1D performance) [42]. This demonstrates how adiabat should be considered in the future if this new regime is investigated in further detail.

The second key class of instabilities observed in ICF reactions are parametric instabilities, which instead arise from laser-plasma interactions. These include stimulated Raman scattering (SRS), stimulated Brillouin scattering (SBS), filamentation and two-plasmon decay (TPD) [45]. Such instabilities result in the reduced coupling of drive energy and the generation of hot electrons, which contribute to preheating of the fuel and reduced yield [46]. This work aims to minimize these instabilities by limiting the peak laser power in order to keep *Iλ*^2^, the product of intensity and the square of wavelength, below $10^{14}\;({\text{W cm}}^{-2})\;\text{μ}{\text{m}}^{2}$, a threshold value above which significant onset of these instabilities is observed [47]. The laser wavelength in this work is kept constant at 351 nm, making this effectively a limit on intensity.

## 3. Simulation details

Simulations were performed using the 1D radiation hydrodynamics code HYADES [48]. Details of benchmarking simulations can be found in appendix A. To simulate ICF experiments, a 1D spherically symmetric geometry was used. HYADES uses a Lagrangian framework for modelling hydrodynamic processes, and requires the user to mesh the problem. A custom meshing algorithm was produced, ensuring that the zone thickness at the outer capsule edge remained below 0.01 μm and that the relative mass difference between successive zones was kept below 0.02. Resolution of this order is required to ensure meaningful results. A multi-group radiation diffusion approximation was used to describe radiation transport, using 70 radiation groups. An average-atom ionization model was used. Electron flux limiting of 0.05 was used, while an ion flux limiter of 0.4 was applied. A multiplier of 0.8 was applied to the input laser energy to account for losses from cross-beam energy transfer (where energy is transferred between incoming laser beams through an intermediary plasma wave, which results in a significant reduction in energy reaching the target [49]), and refraction.

**Figure 1.**
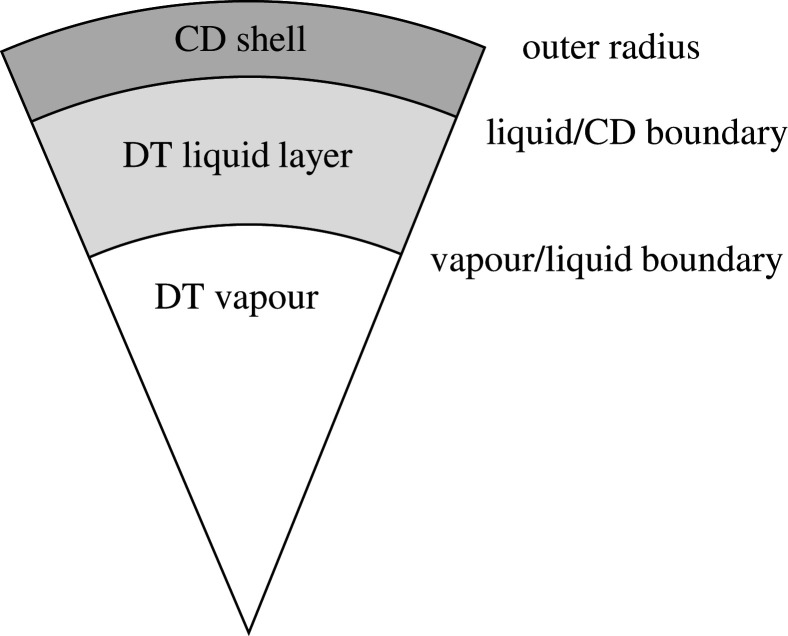
Section of a schematic capsule diagram, showing the layer composition and the different boundaries.

The capsule consisted of a central region of DT vapour, surrounded by a DT wetted foam layer and a thin deuterated plastic (CD) shell (as seen in figure 1). The quotidian equation of state (QEOS) model was used to describe the two DT regions, while the SESAME EOS table was used for the CD layer. The density of the DT vapour was varied between 0.65 mg cm^−3^ and 1.05 mg cm^−3^. These vapour densities require temperatures above the melting point of DT ice, but are within the range tolerated by liquid and wetted foam layers [5].

As a 1D hydrodynamic code, HYADES simulates neither parametric nor hydrodynamic instabilities. In order to ensure physically meaningful results, it is therefore important to ensure that such instabilities are minimized. This is achieved in this work through the upper limit of 16 applied to convergence ratio, of 30 to IFAR, of 400 km s^−1^ to implosion velocity, and of $10^{14}\;({\text{W cm}}^{-2})\;\text{μ}{\text{m}}^{2}$ for *Iλ*^2^, as explained in §2.

**Figure 2.**
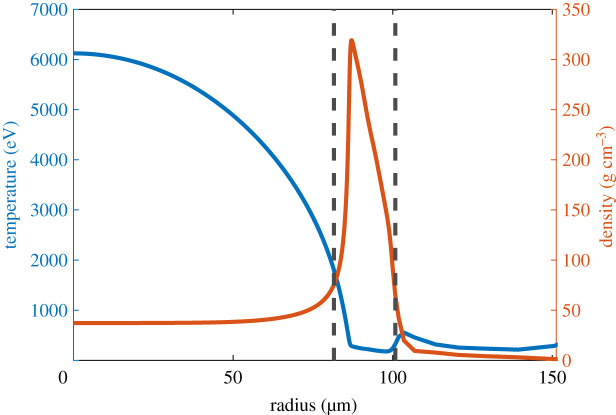
Density (orange/grey) and ion temperature (blue/black) in the centre of a capsule with 1.425 mm outer radius, at the time of maximum compression. The left dashed line indicates the boundary between the hotspot and the shell, while the right dashed line indicates the shell outer boundary.(Online version in colour.)

The measurements of convergence ratio, IFAR, and implosion velocity all depend upon the definition used for the hotspot and shell; despite this, there is no universally agreed definition for these terms. An example plot of density and ion temperature at the time of maximum compression can be seen in figure 2. The hotspot is roughly defined as the hot region of low density at the centre of the capsule, surrounded by the high-density and low-temperature shell. However, the transition between the two is continuous (as can be seen in figure 2), and different work identifies the exact boundary in different ways. In this paper, the shell outer boundary is defined for each timestep as the radius where the density falls to 1/e^2^ of the peak value. The hotspot/shell boundary is also defined using density, but the definition used for the outer boundary was found not to work as the hotspot density itself could at times be larger than this value. As such, the hotspot boundary was defined as the radius where the difference between the density and the density at the centre is equal to 1/e^2^ of the difference between the peak density and this central density. This definition appears to identify an appropriate boundary, and has the added advantage of working throughout the early stages of the implosion as well as at maximum convergence (unlike definitions based on a constant threshold temperature/density). The positions of these two boundaries are indicated in figure 1.

## 4. Simulation campaign

A simulation campaign consisting of over 10 000 simulations was performed, of which over 6000 fit the criteria for implosion velocity, convergence ratio and IFAR. The intention of this was to produce pulse sequences and capsule designs capable of producing high neutron yields and gains at a number of different energies. While attempts have been made to maximize the gain as much as possible for all energies and sequences reported on, the number of variables in this problem mean that no claim can be made that they are optimal. As such, it is recommended that a more comprehensive optimization campaign is undertaken using machine learning techniques such as those applied by Hatfield *et al.* [50], in order to better explore and understand this parameter space. The focus of this work is to demonstrate that high gain can be achieved in this regime, rather than to provide an exhaustive search of parameters to find the maximum gain to a given accuracy.

As capsule size increases, the laser energy required for the implosion also increases. This is driven by two effects: the laser is applied for longer as the capsule takes longer to implode, and the larger capsule size permits a larger power to be used while maintaining the same intensity (and thus *Iλ*^2^). An initial capsule was first simulated and optimized, with an outer radius of 2.85 mm. Further capsules were then produced by multiplying this outer radius by different scaling factors. Five different capsule sizes were optimized in total, corresponding to five different energy scales. These had radii of 1.4250, 1.8525, 2.4225, 2.8500 and 3.1350 mm, corresponding to size multipliers of 0.5, 0.65, 0.85, 1 and 1.1, respectively. In order to maintain a constant intensity and thus satisfy the *Iλ*^2^ constraint, the laser power was multiplied by the square of the size multiplier. This resulted in a constant intensity of 6.8 × 10^14^ (W cm^−2^) for all capsules, and a constant *Iλ*^2^ of $8.4\times 10^{13}\;({\text{W cm}}^{-2})\;\text{μ}{\text{m}}^{2}$ (which satisfies the aforementioned criterion).

**Figure 3.**
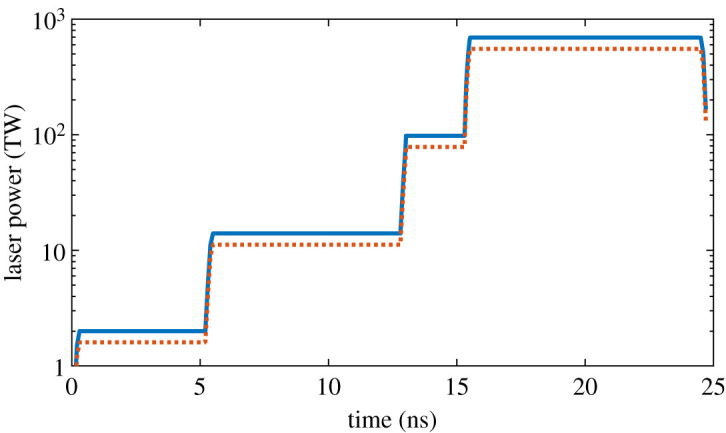
The 4 pulse laser profile used for the 2.85 mm capsule, with pulse powers of 2, 14, 98 and 692 TW. The total energy is 6.73 MJ. The input laser power is displayed in blue/black, while the orange/grey line shows the power applied to the capsule (once a 20% reduction is applied to account for cross beam energy transfer and refraction).(Online version in colour.)

A stepped laser profile was used in each case, corresponding to a series of stacked trapezoids. The steps are referred to as pulses, so that a laser profile with three regions of increasing laser power (each followed by a period of constant power) is referred to as a 3-pulse sequence. The optimized 4-pulse sequence for the 2.85 mm capsule can be seen in figure 3. Each pulse has a rise and fall time of 0.2 ns, and there is no decrease in laser power until the laser is switched off at the end of the sequence. The pulse sequences were designed following the theory described by Lindl for creating low-adiabat laser profiles [19]. For a pulse sequence to give a low adiabat two things are required: the shock produced by the first pulse should not be too large, and the relative increase in pressure between shocks should be constant. The 3-pulse sequence proved reasonably optimal with regards to the relative pressure increase, and alternative pulse sequences attempted with a lower first pulse amplitude did not improve performance. For the 4-pulse sequence, a laser profile was designed to give a constant ratio between successive pulse powers, and the power of the first pulse was also designed to be low. The 3-pulse sequence had relative pulse powers of 24, 215 and 692, while the 4-pulse sequence had relative pulse powers of 2, 14, 98 and 692 (these pulse powers quoted are not additive—the total power applied is 692, and not 806). These pulse powers appeared to be reasonably well optimized; however, a study aiming to explore the multi-dimensional parameter space more comprehensively certainly may find other amplitudes that offer some degree of improvement.

The optimization of the capsule layer thicknesses and the laser timings is an inexact process. It was found during the course of the simulation campaign that the optimal pulse timings roughly followed a similar pattern, and so this was used to provide a first estimate of the initial pulse sequences. For the 3-pulse sequence, this meant timing the pulses so that the shocks from the first and second pulse coalesce just as they break through from the liquid layer into the vapour, with the third pulse applied just as this occurred. Having shocks coalesce in this way is expected to minimize entropy injected into the fuel, and as such to minimize the adiabat [19]. In the case of the 4-pulse sequence, the pulses were timed so that the first and second shocks coalesced soon after breaking through from the CD into the liquid layer. The third shock was then timed to coalesce with the now combined first and second shocks just as they break into the vapour, with the fourth pulse applied at roughly this time. With this initial pulse sequence produced, parameter scans were then performed over the pulse timings. This was done until a pulse sequence offering a good combination of gain, CR, implosion velocity and IFAR was found. Parameter scans were then performed over the layer thicknesses using this sequence (varying the vapour/liquid and liquid/CD boundaries, so that the outer radius was kept constant), for a range of different vapour pressures (controlled through the vapour density). The simulation offering the best gain for the allowed CR, implosion velocity and IFAR values was again selected. The laser parameters were then re-optimized for the new capsule thicknesses. Finally, the laser switch off time was varied, and the optimal selected—this was then accepted as the overall optimal simulation for the capsule. It is clear that this process is rather subjective and does not well capture the multidimensional nature of the problem, but it proved effective in increasing the gain and for providing an estimate of what could potentially be achieved in this regime.

## 5. Results

**Figure 4.**
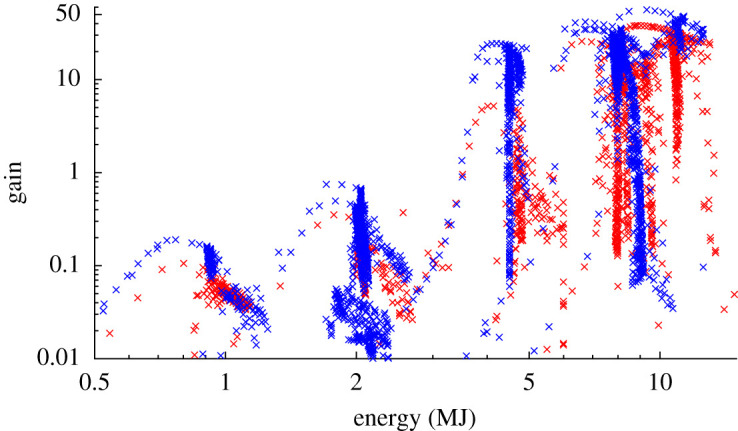
A log-log plot showing the simulated gain vs input energy, for the simulations where the criteria to minimize instabilities were satisfied. Simulations where a 3-pulse sequence was used are represented by a red/grey point, while those using 4-pulse sequences are coloured blue/black. 5 groupings of points are visible, corresponding to the five different capsule sizes used.(Online version in colour.)

Figure 4 displays the simulations that fit the required criteria on a log-log plot of gain versus laser energy. A rough grouping of the points into 5 peaks can be seen; this corresponds to the five different capsule sizes, and thus five different energy scales under consideration. The 3-pulse and 4-pulse sequences are displayed in red/grey and blue/black respectively, and it can be seen that in each case the 4-pulse sequence offers a significant increase in gain. This is to be expected, as 4-pulse sequences result in more isentropic compression and lower fuel adiabats. Both sequences were optimized for each of the five capsule sizes. Details of the optimal simulation for all capsule sizes and both pulse sequences are given in table 1. The increase in gain between the optimal 3-pulse and 4-pulse sequence for each capsule size ranged between 42% and 360%. The variance in this increase is likely due to the fact that the relationship between input energy and gain varies with adiabat. Ignition will occur at lower energies for lower adiabats, and so energies near the ignition threshold will see a larger increase in gain between the two pulse sequences (such as that observed at 4.2 MJ).

**Table 1.** Simulation parameters used to achieve the maximum gains for each capsule size for both pulse sequences. The original capsule was the column with size multiplier of 1. New capsule sizes (and thus energy scales) were simulated by multiplying the radius by the size multiplier. In order to keep *Iλ*^2^ constant over different capsule sizes, the pulse powers were then multiplied by the square of the size multiplier. Gain is given to two significant figures, while convergence ratio, IFAR and implosion velocity are given to one decimal place. This level of precision is higher than would typically be quoted for a 1D simulation, but is used here to indicate how the values compare with the imposed limits. While convergence ratio is in places displayed as 16.0, the value to the precision calculated in the analysis is below this upper limit.

number of pulses size multiplier	3				
	0.5	0.65	0.85	1	1.1
laser energy (MJ)	0.801	1.78	4.01	6.61	8.88
gain	0.11	0.35	5.2	26	38
convergence ratio	15.3	15.5	15.9	16.0	16.0
IFAR	20.3	18.7	15.2	14.3	12.7
implosion velocity (km s^−1^)	399.2	395.4	373.9	358.4	332.4
max pulse power (TW)	173.00	292.38	500.12	692.13	837.50
Pulse 2 switch on time (ns)	3.65	3.80	5.00	6.20	7.00
Pulse 3 switch on time (ns)	4.15	4.90	6.30	7.60	8.80
laser switch off time (ns)	8.50	10.50	13.75	16.50	18.60
vapour/liquid boundary (mm)	1.3000	1.6835	2.2100	2.6100	2.8710
liquid/CD boundary (mm)	1.4000	1.8200	2.3800	2.7900	3.0580
outer radius (mm)	1.4250	1.8525	2.4225	2.8500	3.1350
vapour density (mg cm^−3^)	1.00	0.85	0.65	0.65	0.70

number of pulses size multiplier	4				
	0.5	0.65	0.85	1	1.1
laser energy (MJ)	0.768	1.71	4.21	6.73	8.48
gain	0.19	0.75	24	42	54
convergence ratio	16.0	15.8	15.9	16.0	15.7
IFAR	29.7	25.1	21.2	18.4	15.7
implosion velocity (km s^−1^)	399.6	399.6	347.5	334.1	311.0
max pulse power (TW)	173.00	292.38	500.12	692.13	837.50
Pulse 2 switch on time (ns)	2.60	3.60	3.70	5.20	5.70
Pulse 3 switch on time (ns)	5.60	7.80	9.40	12.80	15.00
Pulse 4 switch on time (ns)	6.80	9.50	11.40	15.30	18.00
laser switch off time (ns)	11.00	15.00	19.40	24.50	27.50
vapour/liquid boundary (mm)	1.3050	1.6705	2.2100	2.6000	2.8270
liquid/CD boundary (mm)	1.3950	1.8200	2.3630	2.7800	3.0580
outer radius (mm)	1.4250	1.8525	2.4225	2.8500	3.1350
vapour density (mg cm^−3^)	1.05	1.00	0.85	1.00	1.00

It is clear from table 1 that high gain can be achieved in this regime over a range of energy scales, despite the stringent restrictions discussed in §2. The gain of 54 achieved at 8.5 MJ, while at a much higher energy than currently achievable experimentally, is a significant result. This is the level of gain commonly acknowledged to be required for an economically viable fusion reactor [51]. The gains of 20 and 41 achieved at 4.2 MJ and 6.7 MJ, respectively, also demonstrate how high gain can be achieved at more moderate energies (although still much higher than current facilities). The lower energy (800 kJ and 1.7 MJ) simulations serve to demonstrate the viability of this approach on current facilities. Both of these results are greater than any yield or gain previously achieved in an ICF experiment [52], clearly indicating the potential of this technique. In the case of the 1.7 MJ result, this is greater by an order of magnitude. These represent (to our knowledge) the first simulations of liquid layer/wetted foam capsules in a direct-drive configuration, and suggest significantly greater yields than previously achieved in liquid layer implosions (4.5 × 10^14^) [5]. It is also worth noting the lower IFAR of the 3-pulse sequences; previous work has suggested that an IFAR limit lower than 30 may be required [42,43], and so the lower IFAR 3-pulse sequences offer an indication of the performance that could be achieved if this was necessary.

While the simulations presented here use powers and energies equivalent to Laser Mégajoule (LMJ) and the NIF, it is not currently possible to perform such experiments on these facilities. Such facilities are designed for indirect drive, with the lasers positioned at the poles of the capsule; this is incompatible with direct-drive experiments, where uniform compression would require the lasers to be distributed more evenly. One potential solution that would allow direct-drive experiments to be performed on these facilities is polar direct drive (PDD) [53]. In this technique, the lasers are confined to the poles of the capsule, and the pointing and power of the individual beams are then individually adjusted to attempt to provide uniform compression. The polar laser placement makes this approach much more compatible with indirect drive facilities [54], and could therefore offer a way to enable the experiments proposed in this work to be performed on LMJ or the NIF. However, these facilities are not fully equipped to perform a high gain PDD experiment with cryogenic capsules at the current time; at the NIF, for example, such an experiment would require the development of a PDD compatible cryogenic platform and bespoke phase plates [55]. In addition to this, the PDD approach itself has issues that may prevent successful high-gain experiments. Cross-beam energy transfer is particularly significant in PDD, and can result in large amounts of scattering and significantly reduce the energy that reaches the target [56]. There are also problems with uniformity, leading to proposals such as ‘saturn’ capsules to try and account for the reduced drive at the equator [54]. This all contributes to the findings in appendix A, where it was found that a lower input multiplier (and thus higher powers/laser energies) are needed to describe PDD implosions. The simulations here best describe symmetric direct-drive experiments, and so a reduction in performance would be expected for a PDD experiment based on one of these designs. It is clear that experiments to verify this work would need to occur in a PDD configuration if they were to be performed on the current facilities. However, it is also clear that a significant amount of work remains to be done before this would be possible.

The NIF-scale (1.7 MJ) result is particularly interesting. At this energy, the capsule appears to be right on the cusp of ignition, with dramatic increases in yield and gain with implosion velocity (the implosion velocity appears to be the limiting factor for the smaller capsules, with larger capsules seeing lower velocities which fit more comfortably below the 400 km s^−1^ limit). As such, it is of interest to see what level of performance could be achieved if the limit on implosion velocity is relaxed. It was found that allowing implosion velocity to increase up to 414.9 km s^−1^ enabled a gain of 1.12; this suggests the possibility of net gain on the NIF. The velocity limit chosen in §2c was chosen to represent realistic capsules, and is not a specific value with a strong theoretical or experimental grounding. However, this upper limit is already quite high; experiments on the NIF have demonstrated implosion velocities of over 370 km s^−1^ (towards this upper limit) with no evidence of mixing [57], but a velocity of 400 km s^−1^ would already require a reasonable improvement. Further increasing the velocity therefore carries significant risk, and so this 414.9 km s^−1^ simulation should serve only to demonstrate the proximity of the NIF scale simulations to the break-even threshold.

**Figure 5.**
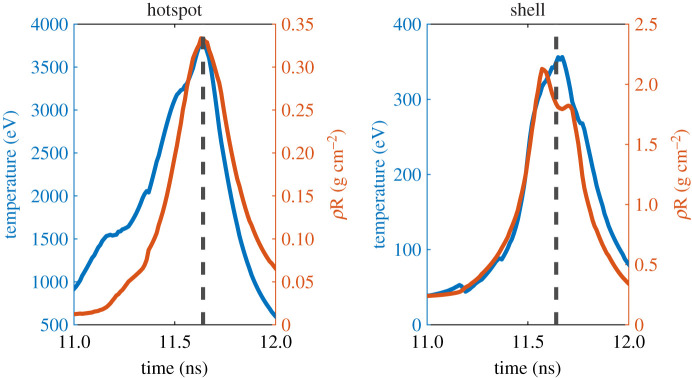
Plots displaying the mass-averaged ion temperature (blue/black) and mass-averaged areal density (orange/grey) in both the hotspot and the shell for the 4-pulse LMJ-scale implosion, covering the time period where fusion occurs. The black dashed line indicates the neutron bang time (the time at which maximum neutron production occurs).(Online version in colour.)

The LMJ-scale (800 kJ) capsule is a promising candidate for further study. This simulation offers the prospect of record yield and gain for ICF experiments, without having to operate at such extreme energies. The opportunity to not be reliant on a single facility is of major benefit, as is the reduced risk of damage to equipment from operating at lower energies. Figure 5 shows the ion temperature and density in the hotspot and shell of the LMJ scale capsule, over the time period where fusion occurs. It can be seen in this figure that the hotspot *ρR* is greater than 0.3 g cm^−2^, while the ion temperature is around 3.5 keV. The *ρR* in the shell is around 2 g cm^−2^ (and the ion temperature is much lower at around 350 eV, as expected). The hotspot areal density in the LMJ-scale simulation is notable, as 0.3 g cm^−2^ is sufficient for ignition and matches the target areal density for the NIC ignition design [24]. However, actually igniting such a capsule requires higher temperatures; the analytic model discussed in §2 estimates that such an areal density would require an ion temperature of around 5.5 keV to ignite, which is in agreement with the temperatures targeted during the NIC from hydrodynamic assembly (it was expected that the ultimate temperature for such a capsule would then reach 10–12 keV, since significant self-heating would occur once ignition took place) [1].

These specific results themselves are not particularly impressive—it is the fact that such results have been achieved in this new low-instability regime which is of note. Much higher gains than those quoted here have been reported at lower energies in previous simulation work. The novel aspect of this work is that such results have been achieved in this new regime, where instabilities are expected to be minimized. The expected 1D nature of implosions in this regime means that, while the gain itself is not high for 1D simulations, there is good reason to expect closer agreement between experiment and 1D simulations than is typical. It is therefore hoped that gains of similar magnitude to those presented here could be achieved experimentally, which is not true of typical high gain 1D simulations.

In addition, it is important to note that the use of 1D simulations means that high accuracy with these results is not claimed. It is expected that behaviour in this regime would be close to 1D-like, but more detailed simulations would be required to make exact claims about performance. Instead, these results are intended as indicators of the level of performance that may be possible in such a regime, to assess its viability. The potential of stable implosions in this regime, coupled with the fact that these estimates of gain are indeed high enough to be of interest, suggests that this regime does therefore warrant further investigation.

## 6. Outlook

The results demonstrated in this paper suggest some clear directions for future work. The most obvious next step is to increase the dimensionality, performing 2D simulations to investigate hydrodynamic instability growth and provide further confidence that performance is approximately one-dimensional in this regime. Such simulations would also provide a more accurate estimate of the performance that could be expected from such capsules (particularly as the agreement observed with experiment in [6] was found using 2D simulations). An investigation of this regime placing limits on the adiabat would also be an interesting extension of this campaign. Work is ongoing to investigate the possibility of room temperature capsules using foam layers with the same total density, but without DT wetting. Such a capsule would allow the hydrodynamics of the implosion (and the assumptions about 1D performance) to be tested, without significant neutron generation or the need for a cryogenic platform (akin to work using symmetry capsules to study hydrodynamic behaviour [58]). It is anticipated that such an experiment could be performed in a PDD configuration on LMJ or the NIF.

The promising areal density but sub-ignition temperatures demonstrated in figure 5 suggest that the LMJ scale capsule would be a promising candidate for auxiliary heating. This could potentially be achieved using a scheme such as the overlapping relativistic electron beams proposed by Ratan *et al.* [8]. Using this approach, it should be in principle possible to inject a large amount of energy into the hotspot around the time that the peak *ρR* occurs, causing a rapid increase in ion temperature. Figure 5 suggests that if an increase in ion temperature of around 2 keV could be achieved, ignition could potentially be achieved at LMJ-scale energies. This provides another interesting opportunity for future research, and simulations are ongoing to investigate the feasibility of such an approach.

For future possible experimental verification of this work to occur, continued development of key technologies is required. This work adds further justification to the interest in these approaches. Currently, there is no capability for performing direct-drive shots of cryogenic targets at the NIF; the development of a cryogenic polar direct-drive platform and bespoke phase plates would enable this and therefore greatly benefit direct-drive research. Continued development of capsule manufacturing techniques would also be welcomed. As mentioned in §1, the CH foam densities that are currently achievable for wetted foam capsules are substantially greater than the 10 mg cm^−3^ approximate upper limit for which the pure DT EOS used in this work is a good model. This places a major limitation on simulation work using this approach for wetted foams, and the development of techniques enabling foams of this density would be extremely beneficial. Alternatively, the availability of EOS and opacity data describing existing wetted foams would allow simulations to be performed that would show the performance that could be achieved using current technologies. This would also be a useful development.

## 7. Conclusion

A simulation campaign consisting of over 10 000 simulations was performed, seeking to simulate ICF implosions at a range of relevant energies. Convergence ratio, IFAR and implosion velocity were restricted in order to ensure realistic implosions and minimize hydrodynamic instabilities, while peak laser power was limited to keep *Iλ*^2^ underneath the threshold for parametric instabilities. Doing so ensured that the 1D simulations should remain physically meaningful, and show good agreement with experiment.

It was found that gains of 0.19 and 0.75 could be achieved at LMJ and NIF energy scales, respectively, both of which would represent record gains and yields for ICF experiments. The gain at NIF-scale energies can be increased to 1.1 if the restrictions on implosion velocity are relaxed slightly, raising the possibility of break-even on current facilities. Further increases in gain were observed for increasing energies, culminating in an achieved gain of 52 for an 8.5 MJ implosion. While the use of 1D simulations means that the specific values quoted should not be considered as highly accurate, these results do suggest that the low-instability growth regime identified could potentially offer sufficiently high gains to be of interest for fusion energy purposes, and that further investigation of this regime is warranted.

2D simulations are therefore planned to further investigate this regime, and to test the assumptions of 1D-like performance. Work is also ongoing to investigate the use of room temperature foam capsules to study the hydrodynamics of the capsules simulated in this work, which could hopefully be used in the future for experimental verification that this regime minimizes instability growth. Further work is also encouraged in developing capsule manufacturing to enable lower density CH foams to be produced. Finally, it was identified that the areal densities simulated in the hotspot of the LMJ scale implosion of 0.3 g cm^−2^ could be sufficient for ignition, were the ion temperature of 3.5 keV to be raised using auxiliary heating. Future simulations are planned to investigate the possibility of applying such techniques to lower the energy required to achieve ignition/reactor level performance in this regime, in the hopes of achieving high gains at viable energies for fusion power.

## Figures and Tables

**Figure 6.**
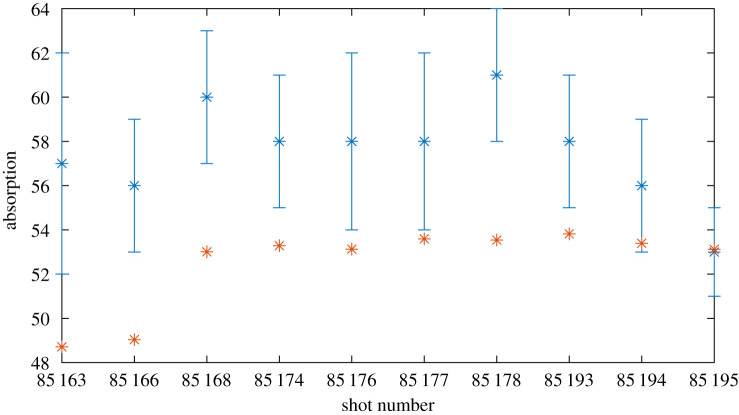
Fractional absorption of the laser energy. The orange/grey points are the simulated data, while the blue/black points are the experimental data. The simulated data is obtained by measuring the absorption within the simulation, and multiplying this value by the 0.8 input power multiplier used to account for CBET.(Online version in colour.)

**Figure 7.**
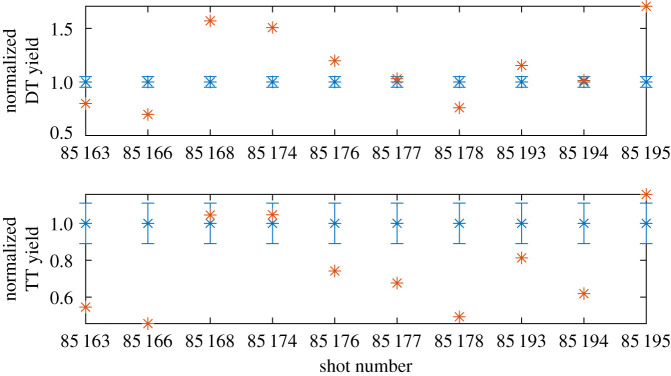
DT and TT yields for the different OMEGA shots, normalized to the experimental data. The orange/grey points are the normalized simulated data, while the blue/black points with a consistent value of 1 are the normalized experimental data, included to show the error bars.(Online version in colour.)

**Figure 8.**
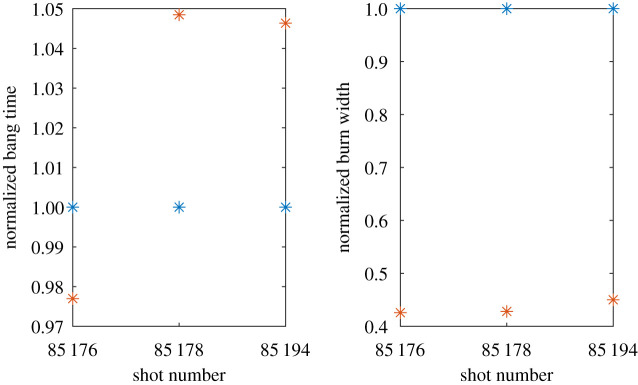
Bang time and burn width for the different OMEGA shots, normalized to the experimental data. The orange/grey points are the normalized simulated data, while the blue/black points with a consistent value of 1 are the normalized experimental data.(Online version in colour.)

**Figure 9.**
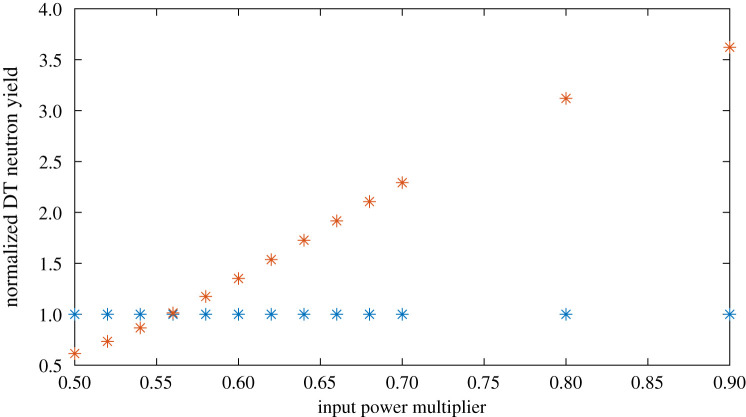
DT yield for NIF shot N190227 simulated using a range of input power multipliers, normalized to the experimental value of 1.11 × 10^16^. The orange/grey points are the normalized simulated data, while the blue/black points with a consistent value of 1 are the normalized experimental data.(Online version in colour.)

**Figure 10.**
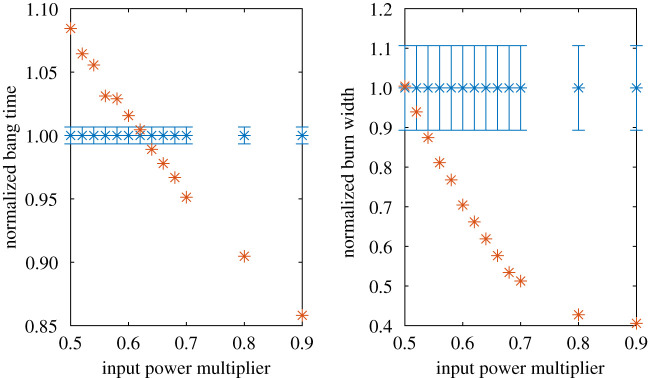
Bang time and burn width for NIF shot N190227 simulated using a range of input power multipliers, normalized to the experimental values of 4.51 ± 0.03 ns and 468 ± 50 ps, respectively. The orange/grey points are the normalized simulated data, while the blue/black points with a consistent value of 1 are the normalized experimental data.(Online version in colour.)

## Data accessibility

This article has no additional data.

## Appendix A. Benchmarking

In order to verify that the simulations performed give reasonable estimates of direct-drive experimental performance, the simulation code and settings used were benchmarked by simulating previously performed direct-drive experiments. This benchmarking was not intended to be exhaustive, as the results presented in this paper are only meant to be taken as an indication of the performance possible in a new regime.

Ten calibration shots performed in a direct-drive configuration on OMEGA for a study of diffusion-dominated mixing were simulated [59]. The capsules used in these shots consisted of different HT vapour mixtures, surrounded by either pure CH or CH with 2% D shells (these shells were homogeneous, and did not have a layer structure such as those presented in the paper referenced). Each of the ten shots was simulated using the parameters described in §3, including the 0.8 multiplier to the laser power used to account for CBET. For each of the 10 experimental shots the laser energy absorbed, DT and TT yields were measured, while the bang time and burn width were recorded for three of the shots.

Figure 6 shows the fractional laser absorption. It is clear that the simulated absorption is typically just outside of the error of the experimental absorption, giving a slightly lower value. This could be improved by increasing the value of the CBET multiplier, but given the reasonable agreement between the two (and the fact that high accuracy is not expected or required from this 1D code) it was decided that this constituted an acceptable level of agreement. Figure 7 shows the simulated DT and TT yields, normalized to the experimental data, for each of the 10 shots. The normalized DT yield has a mean of 1.14 and varies between approximately 0.7 and 1.7, while the normalized TT yield has a mean of 0.76 and varies between approximately 0.4 and 1.2. Again, given that HYADES is a 1D code, this is a reasonable level of agreement for the purposes of this paper, suggesting that the code and settings used are sufficient to give a reasonable estimate of the obtainable yield. It is notable that while the DT yield is a slight overestimate, the TT yield is a significant underestimate. This could be explained by kinetic effects such as fuel stratification [60], which are not included in hydrodynamic codes and are known to lead to increased TT yields compared to when this effect is not modelled [61].

Figure 8 shows the normalized bang time and burn width. The bang time displays very good agreement, varying between approximately 0.97 and 1.05. The burn width is significantly underestimated, with all simulated values being around 0.4 to 0.5 times that of the experimental data. The disagreement in burn width is unexplained, but is likely due to a difference between the definition used in simulating it (the FWHM of neutron rate) and in the experiment (which was unknown to the authors of this work). Given the success of the code in simulating the more important parameters (particularly the DT yield and bang time), it was decided that these results were satisfactory to show that HYADES could be used with these settings to simulate direct-drive implosions.

The polar direct drive NIF shot N190227 [62] was also simulated. This capsule had a 3943 μm outer diameter and a 25 μm CH ablator, and was filled with 6000 torr DT (64% D, 36%T). The laser ramped linearly from 0 to 400 TW over 1.5 ns, remained at this power for 2 ns, and then decreased to 0 TW over 0.5 ns. The simulation parameters were unchanged from those used for the OMEGA shots. A range of input power multipliers were used, as the absorption was expected to be lower due to the PDD configuration. This shot produced a DT yield of 1.11 × 10^16^ neutrons (no error was given for this value), a bang time of 4.51 ± 0.03 ns, and a burn width of 468 ± 50 ps.

The normalized DT yield for the different input power multipliers can be seen in figure 9. Reasonable agreement is seen for lower value input multipliers, with the best agreement observed for multipliers between 0.54 and 0.58. The multiplier of 0.8 used for the OMEGA work gives a DT yield for the NIF shot approximately three times higher than that measured experimentally. The normalized bang time and burn width can be seen in figure 10. The normalized bang time shows good agreement (between 1.05 and 0.95) for input multipliers ranging from 0.56 to 0.7, although it is closest for around 0.62. The burn width is within the error for an input multiplier of 0.5 to 0.52, and is approximately 0.7 times that of the experimental value for a multiplier of 0.6. These three sets of results together suggest that good agreement can be observed for input multipliers of around 0.55 to 0.6, which is significantly lower than the 0.8 used in the OMEGA simulations.

The requirement for a lower multiplier can most likely be explained by the PDD configuration. As discussed in §5, PDD is particularly susceptible to cross beam energy transfer, which would result in less of the laser energy being absorbed. In addition, as the capsule is compressed, the lasers pointing at the equator in a PDD configuration will no longer be incident on the target, resulting in a fraction of the laser energy not being absorbed. This effect will be most significant at later times where the capsule radius is smallest, which is also when the maximum power is applied. This likely explains the need for a lower multiplier, and so it is worth bearing in mind that the 0.8 multiplier used in the simulation campaign is applicable for a symmetric direct-drive configuration, but reduced performance should be expected if a PDD configuration is used instead (or that an input power around 30% higher would be needed to achieve equivalent performance, along with the associated increase in energy).

These results appear sufficient to suggest that HYADES can be used to gain a reasonable indication of the performance for direct-drive implosions, as it is used for in this paper. This benchmarking is not exhaustive, and could be expanded by looking at more complicated capsule designs. In particular, simulations involving wetted foam capsules would be useful, as this would give some indication of how significant the presence of the CH foam is, and increased confidence in the results. As this paper considers only 1D simulations (and uses them only for an initial exploration of a new regime), it was decided that the benchmarking seen here was satisfactory to demonstrate that HYADES can be used for this application.
